# Year 2023 in Biomedical Natural Language Processing: a Tribute to Large Language Models and Generative AI

**DOI:** 10.1055/s-0044-1800751

**Published:** 2025-04-08

**Authors:** Cyril Grouin, Natalia Grabar

**Affiliations:** 1 Université Paris Saclay, CNRS, LISN, 91400 Orsay, France; 2 UMR8163 STL, CNRS, Université de Lille, Domaine du Pont-de-bois, 59653 Villeneuve-d'Ascq cedex, France

**Keywords:** Biomedical Natural Language Processing, Topics, Issues, Best Papers, 2023

## Abstract

**Objectives** : This synopsis gives insights into scientific publications from 2023 in Natural Language Processing for the biomedical domain. We present the process we followed to identify candidates for NLP's best papers and the two best papers of this year. We also analyze the current trends in the 2023 publications.

**Methods** : We queried two bibliographic databases (Medline and the ACL anthology) and refined the outputs through automatic scoring. We then manually shortlisted publications to review and selected candidate papers through an adjudication process. External reviewers assessed the interest of the 13 selected candidates. At last, the section editors chose the best NLP papers.

**Results** : We collected 2,148 papers published in 2023, of which two were the best and selected as part of this NLP synopsis. Both address language models and propose solutions for data augmenta-tion, domain-specific model adaptation, and model distillation. Work is done on social media con-tent and electronic health records, using deep learning approaches such as ChatGPT and large lan-guage models.

**Conclusion** : Trends from 2023 cover classical NLP tasks (information extraction, text categoriza-tion, sentiment analysis), existing topics from several years (medical education), mainstream applications (Chatbots, generative approaches), and specific issues (cancer, COVID-19, mental health). Specifically for COVID-19, current researches deal with post-COVID-19 conditions, and they explore the understanding of how this pandemic has been managed and welcomed by populations. In addition, due to language models, a few works have been done to process languages other than English, especially using language portability approaches.

## 1. Introduction

Natural Language Processing (NLP) aims to provide methods, tools, and resources designed to mine textual and narrative documents and enable access to the information they convey [ 1 ]. While human languages are complex (for example, learning a human language requires many years for a human to be fluent), the importance of using NLP approaches to mine documents produced by humans has been pointed out for a long time [ 2 ]. In this synopsis, we present the selection process applied this year and then analyze some publications' content.

## 2. Best papers selection process

### 2.1. Automatic extraction and scoring

#### 2.1.1. Description

To identify the papers published during the year 2023 in the field of NLP for the biomedical domain, we queried two bibliographic databases: Medline 1 , specifically dedicated to the biomedical domain, and the ACL anthology 2 , a database that brings together the major NLP conferences (ACL, COLING, EMNLP, LREC, NAACL, etc.) and journals, since some NLP studies concerning the biomedical domain are published in ACL conferences and journals that PubMed does not index.

**Figure 1.**
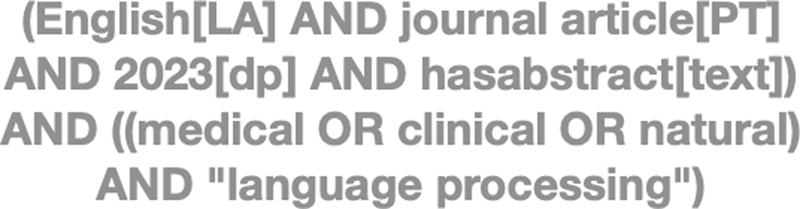
Query used on PubMed for collecting candidate publications for review.

The query we used ( Figure 1 ) is voluntarily basic to bring back the maximum number of candidates. It consists of searching for all papers published in English in 2023, having an abstract, and indexed with 75 terms of clinical language processing, medical language processing, or natural language processing. As of 2023 January 17 ^th^ , we collected 2,114 entries, much more than the two last years (1,204 entries in 2021 and 1,670 entries in 2022). We applied a similar query to the ACL anthology database and collected 34 additional entries. To refine the selection of the candidate's best papers, we defined an automatic scoring for each candidate based on five criteria (journal name, objectives, methods/corpus/resources used in the paper, evaluation/metrics used, particular concepts or key phrases used in the abstract). We aim to identify scientific papers valuable for the NLP community and discard medical papers that apply existing NLP methods without any valuable improvement. For example, the “language” concept may highlight the object study, which concerns NLP and computer science, or a cognitive aspect with language disabilities, which concerns neuroscience and psychiatry.

**Figure 2.**
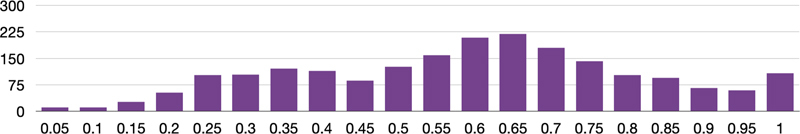
Distribution of papers according to the filter scores.

We also excluded yearbooks and survey papers since they do not fit the criteria for best paper candidates. To focus on original contributions, we gave a lower score to abstracts that specifically mention phrases like “using natural language processing” or “perform a natural language processing analysis” since the NLP dimension is not central in those submissions. Consequently, those papers are not good candidates for the best papers in NLP. For each of the 2,148 candidate papers, the final score ranged from 0 to 1 ( Figure 2 ).

#### 2.1.2. Analysis

**Figure 3.**
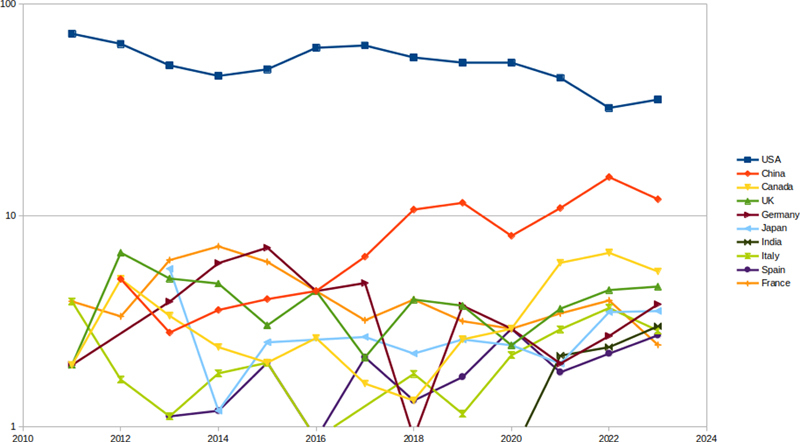
Top-10 first author's affiliation country from 2011 to 2023 in the PubMed extractions sorted by decreasing percentages of publications F(logarithmic scale).

We present in Figure 3 the evolution of the proportion of the first author's affiliation country from 2011 to 2023 in the PubMed export as provided by our query ( Figure 1 ), limited to the ten countries best represented in the publications (U.S., Canada, several Western European countries, India, and Japan). Note that the y-axis is logarithmic to make visible countries with a low proportion of first authors.

Table 1 presents the number of published papers per year for each country of affiliation of the first author, based on the output of the PubMed query we defined previously ( Figure 1 ).

**Table 1.** Number of papers collected in the PubMed database from 2013 to 2023 for each country based on the first author's affiliation country.

Country/Year	2013	2014	2015	2016	2017	2018	2019	2020	2021	2022	2023
Canada	6	4	4	3	3	3	9	12	33	42	40
China	5	6	8	5	12	24	40	34	60	96	88
France	11	12	12	5	6	9	11	12	19	25	18
Germany	7	10	14	5	9	2	13	12	11	17	28
India	0	0	0	0	0	1	2	3	12	15	22
Italy	2	3	4	1	0	4	4	9	16	23	21
Japan	10	2	5	0	5	5	9	10	11	22	26
Spain	2	2	4	1	4	3	6	12	10	14	20
U.K.	9	8	6	5	4	9	13	10	20	28	34
U.S.	92	77	98	71	120	126	184	218	251	206	261

The main findings of this evolution are the following:

- Each country generally publishes more papers each year, and those papers are much more indexed in PubMed ( Table 1 ). This is not visible in the figure since the y-axis shows how a country is represented by its first authors in terms of percentage and not the total number of publications of those first authors;
- Until 2020, the U.S. was concerned by more than half of the publications with a first author affiliated within an institution from the U.S. Yet, starting from 2021, the U.S. becomes represented by the first author in around a third of the published papers. The blue line in the figure is slowly decreasing, representing this lower representation. Conversely, the representation of China in the first authors of published papers is growing each year, and the orange line in the figure is thus increasing and closer to that of the U.S.;
- Since 2021 (post-COVID-19 pandemic), the ranking of the second country (China), third country (Canada), and fourth country (the U.K.) did not vary, while the ranking changed for countries beyond the fifth position.

### 2.2. Human Selection

#### 2.2.1. Pre-selection

Based on titles, keywords, abstract contents, and the automatic scoring, used as additional information to make the process faster, each section editor rapidly reviewed those 2,148 entries. This year, we only processed papers with an automatic score higher than 0.5, allowing us to discard half of the entries that were generally unrelated to NLP topics (see next Section). Each section editor independently classified the entries into three classes: Yes / Maybe / No. We thus collected 86 papers classified into Yes or Maybe classes by at least one section editor. We then performed an adjudication process to choose the final top 13 candidates to be proofread by external reviewers. We paid attention to the topics addressed by the researchers to provide enough diversity. As a result, out of the 13 papers, six come from the U.S., two from South Korea, and one from five other countries (China, Germany, Singapore, Spain, and the U.K.). Based on the external reviews and our reviews, we finally decided to keep only two papers as best for the NLP section.

#### 2.2.2. Final best papers

Both best papers deal with language models, highlighting the difficulty of training language models on the clinical data, especially for large language models (LLMs), due to the difficulty to access those sensitive data. To tackle this issue, Tan *et al.* [ 3 ] propose to use data augmentation techniques to benefit from more available data and prompt engineering to fine-tune existing models for a classification task. While language models are generally heavy, Rohanian *et al.* [ 4 ] explore compacting biomedical models using continual learning of existing models on a PubMed dataset and distillation procedures. The first best paper proposes solutions regarding data and model adaptation to domain-specific data. In contrast, the second paper addresses the models to make them lighter (see Appendix A for a more detailed summary of these two papers).

## 3. Current trends in biomedical NLP

As an introduction, we observed that language models (all BERT-related models) based on text content are currently also used to process sequences of characters that do not compose words but are helpful content to other disciplines. This is true for processing sequences of proteins [ 5 , 6 ] or nucleotides in genomic sequences [ 7 8 9 ]. Second, those language models contributed to their democratization outside the NLP community, making them easily usable for non-specialists who published the results of their experiments in scientific papers. These considerations highlight the ability to transfer NLP techniques to computer-based approaches in biology and medicine. Another major trend we have observed for a few years now is how researchers published and valorized the results of their research. If making available the produced corpora is still complex, the availability of produced language models is effortless through the existing platforms (HuggingFace or GitHub repository).

In what follows, we first analyze the keywords provided by the authors (Section 3.1). Second, we analyze the languages addressed (Section 3.2) and the sources of data exploited (Section 3.3). Then, we distinguish some typical tasks (Section 3.4) and some emerging topics (Section 3.5). Except for the keywords, the analysis is issued from the 200 top citations according to the scores computed automatically.

### 3.1. Main Keywords from Publications

Out of the 2,114 entries obtained while querying PubMed, the two main keywords used to index papers are Natural Language Processing/NLP (914 papers), part of our PubMed query ( Figure 1 ), and Artificial Intelligence/AI (308 papers). The rapid development of language models leads to a move towards AI-based approaches for several NLP tasks. We noticed that other keywords used by the authors to index their submissions refer to three main aspects of their research:

- First, the type of documents they process: Social Media/Twitter/Reddit (133 papers) or Electronic Health Records (EHR) (127 papers). Those two types are well balanced, highlighting that social media are an alternative solution when EHRs are unavailable.
- Second, the methods used in the experiments: Machine Learning (293 papers), Deep Learning (165 papers), ChatGPT (84 papers), Language Models/Transformers (52 papers), BERT (35 papers), LLMs (33 papers), and Long-Short-Term-Memory (11 papers). All methods currently used are statistical approaches, whatever the term refers to . If regular expressions are used, they are no longer expressed in the keywords list.
- Third, the topics or purpose addressed in the paper: Sentiment analysis/Text Mining/Text Classification, Chatbot, Medical Education, COVID-19, Cancer, Mental Health/Depression/Dementia. It should be no surprise that COVID-19 is still being studied in 2023. Research in this area is no longer focused on the search for treatment but on retrospective studies of the efficacy of vaccines and vaccination hesitancy [ 10 11 12 13 ] and post-COVID-19 conditions [ 14 , 15 ], such as the impact on mental health [ 16 ]. More confidential research focuses on analyzing vaccine-related messages produced by specific laboratories and how these laboratories have managed their communications [ 17 ]. At last, research during COVID-19 is now helpful for re-use for more general medical applications, such as Chatbots [ 18 , 19 ].

### 3.2. Languages Addressed

As in previous years, we observed that Chinese is still the first other-than-English language addressed in NLP publications related to the biomedical domain in 2023. European languages from countries with vast populations and available resources are also well considered: French, German, and Spanish. We hypothesize that countries having few research labs are less represented in terms of processed languages (Dutch, Estonian, Greek, Italian, Norwegian, Swedish). Out of Europe, we found several papers concerning Korean and Japanese languages. The following list is not exhaustive and only reflects a few topics addressed in each language:

- Arabic: sentiment analysis on COVID-19 tweets [ 20 , 21 ];
- Chinese: automatic text classification, named entity recognition, and information extraction tasks are well represented in papers, while traditional Chinese medicine is still a current research topic [ 22 23 24 25 26 27 28 29 30 31 32 33 ];
- Czech, Polish, and Slovak: information extraction in oncology records [ 34 ];
- Dutch: negation identification and language models for a specific clinical issue [ 35 36 37 ];
- Estonian: production of a BERT model for information extraction from Estonian texts [ 38 ];
- Finnish: analysis of social media during the pandemic issue [ 13 ];
- French: language portability of existing algorithms, adaptation of existing models, and information extraction from social media [ 17 , 39 40 41 42 ];
- German: production of corpora, corpus annotation, information extraction, and production of language models [ 43 44 45 46 47 48 ];
- Greek: the issue of COVID-19 long analyzed in social media [ 49 ];
- Italian: information extraction and opinion mining tasks, development of Chatbots, and vaccination hesitancy [ 50 51 52 53 ];
- Japanese: information extraction from social media and reports, use of ChatGPT to generate lists of diagnoses, and production of systems for traditional Kampo medicine [ 54 55 56 57 58 ];
- Korean: information extraction on adverse drug events (ADEs) [ 59 ];
- Moroccan dialect: named entity annotated dataset [ 60 ];
- Norwegian: production of information extraction systems and de-identification system based on related languages [ 61 , 62 ];
- Persian: sentiment analysis from cancer institute feedback forms [ 63 ];
- Portuguese: information extraction and sentiment analysis [ 17 ];
- Russian: production of a dataset from PubMed annotated into nested named entities [ 64 ];
- Spanish: production of resources (corpora, lexicon), information extraction, and langage portability methods to annotate corpora [ 17 , 65 66 67 68 69 70 ];
- Swedish: comparison of a BERT language model and human annotations in an adverse drug reaction triage task [ 71 ].

We observe that solutions are proposed for producing resources and language models, using existing resources from a close language (specifically between Danish, Norwegian, and Swedish, or between Arabic and dialects) or trying language portability, generally from English to another language.

### 3.3. Sources of the Data Processed

As in previous years, NLP researchers work with several sources of data, which have become emblematic of medical research: scientific literature, clinical documents and social media.

One notable difference in 2023 is that the use of clinical data increased importantly. The keywords analyzed also testify this fact. Indeed, several experiments with clinical data addressing a wide variety of tasks have been proposed, such as test of language models for long clinical texts [ 72 ], acquisition of lexicon for the family history [ 73 ], detection of negation and speculation in radiology reports [ 74 ], coreference resolution in clinical narratives [ 75 ], de-identification of radiology reports [ 76 ], rare disease identification from clinical notes [ 77 ], section identification and structuring in clinical narratives [ 68 ], adaptation of LLMs to clinical data through prompting [ 78 ]. Let's also note that radiology reports become again attractive for researchers and are used for instance, for the extraction of entities and spatial relations [ 43 , 79 ], ICD coding [ 80 , 81 ], ADE extraction [ 82 ], patient similarity [ 40 ], or for inferring cancer disease responses from text and images [ 3 ]. In such works, clinical data can be provided from publicly available datasets, like MIMIC-III [ 83 ], or from own datasets with restricted access.

Social media remain another important source of information for researchers and health practitioners. Such data are typically used for the analysis of COVID-19-related issues [ 13 , 20 , 21 , 42 , 49 ], and for mental health [ 54 , 84 85 86 ].

As for the scientific literature, it is exploited in the context of randomized controlled trials (RCTs) and systematic reviews: extraction of evidence for the RCTs [ 87 ], detection of the outcome issues [ 88 89 90 ], automatic assistance for systematic reviews [ 91 , 90 ], detection of high-quality papers [ 92 ]. Besides, scientific literature can also be used in a variety of tasks, such as extraction of relations between suicide and drugs [ 93 ], simplification of scientific abstracts [ 94 ], or the creation of medical lexicon intended to complete the UMLS [ 66 ].

### 3.4. NLP Tasks performed

Among the general NLP tasks, we can mention traditional information extraction, categorization, and prediction. Besides, like in the general NLP area, the wave of generative LLMs has also entered the biomedical domain. Generative models are exploited in a lot of contexts. To provide a few examples, we can mention the extraction of clinical entities and relations [ 88 , 95 ], clinical data augmentation [ 3 , 41 , 76 ], generation of diagnosss lists [ 57 , 56 ], different public health issues [ 96 ], ADE signal extraction [ 97 ], and conversational agents [ 98 ].

Some works investigate specific issues, such as enriching LLMs with biomedical knowledge [ 99 ] or studying the bias [ 100 ].

Besides, as the use of LLMs is well established now, we have noticed several literature reviews, which focus on specific research questions such as the use of ChatGPT in clinical practice [ 101 ], the use of transformers in biomedicine [ 102 ], as well as the use of NLP in emergency rooms [ 103 ], in oncology departments [ 104 ], on radiology reports [ 105 ], for the summarization [ 106 ], or on social media data [ 107 , 108 ].

### 3.5. NLP Research outside the Box

A few topics are addressed but not highlighted in the mainstream research. This is mainly true for literature discovery, where analysis of *known unknowns* reported in the scientific literature was done to produce an ignorance base [ 109 ]. On social media, research is generally done to identify hate and harmful speeches. In contrast, work has been done to explore the characteristics of *peace speech* and to provide an index of peace for each country [ 110 ]. Automatic speech recognition (ASR) systems produce text from speech, enabling the application of conventional NLP approaches to mine information. Nevertheless, information may be expressed by non-lexical conversational sounds such as *Mm-hm* and *Uh-uh* to express positive and negative answers, which have been used to refine ASR outputs from clinical conversations [ 111 ].

## 4. Conclusion

This synopsis paper presents the process we followed to identify the best papers for the NLP section of the IMIA Yearbook. We selected two scientific papers published in biomedical journals. These papers deal with language models and the difficulty of training language models on clinical data. They propose solutions that address data augmentation, domain-specific model adaptation, and model distillation. Unsurprisingly, most biomedical NLP papers published in 2023 deal with deep learning and language models. Consequently, such papers are becoming increasingly numerous. They address classical NLP tasks (information extraction, text categorization, sentiment analysis), existing topics from several years (medical education), mainstream applications (Chatbots, generative approaches), and specific issues (cancer, COVID-19, mental health). Specifically for COVID-19, current researches deal with post-COVID-19 conditions, and they explore the understanding of how this pandemic has been managed and welcomed by populations. Due to the democratization of language models, work is being done to cover more confidential languages ( *e.g.* , Estonian) when producing models, making it possible to process close languages through language transfer methods ( *e.g.* , Moroccan dialect from Arabic). Nevertheless, NLP publications do not cover African languages, and Asian languages are limited to a few (Chinese, Japanese, and Korean).

## Footnotes

1
https://pubmed.ncbi.nlm.nih.gov/

2
https://www.aclweb.org/anthology/
